# Evaluation of MRI Radiomic Features as Surrogates of Tumor Immune Status in Breast Cancer: A Pilot Study of MRI-Derived Radiomic Features for Non-Invasive Immune Profiling in Breast Cancer

**DOI:** 10.3390/cancers18193087

**Published:** 2026-09-23

**Authors:** Mimansha Shah, Soaham Kumar, Narendra Kumar, Jayshree Mishra

**Affiliations:** 1 Irma Lerma Rangel College of Pharmacy, Texas A&M University Health Science Center, Kingsville, TX 78363, USA; 2 Veterans Memorial High School, Corpus Christi, TX 78414, USA

**Keywords:** radiomics, breast cancer, tumor immune microenvironment, CD8+ T cells, machine learning, CIBERSORT, immune score, PyRadiomics, tumor-infiltrating lymphocytes, immunotherapy biomarker

## Abstract

Background: Tumor-infiltrating lymphocytes (TILs) and CD8+ T cell density are important predictors of immunotherapy response in breast cancer, but their evaluation requires invasive tissue biopsies. Radiomics, which enables high-throughput extraction of quantitative imaging features, offers a promising non-invasive approach to characterize the tumor immune microenvironment (TIME). This study evaluates whether MRI-derived radiomic features could predict immune infiltration status in breast cancer. Additionally, we have developed an on-line browser-based tool that can predict the immune status of the tumor environment. Methods: Preoperative MRI and matched transcriptomic data from 38 breast cancer patients were obtained from TCIA and TCGA-BRCA datasets. Tumors were manually segmented using 3D Slicer, and radiomic features were extracted using PyRadiomics. Eighteen biologically relevant radiomic features associated with immune status were selected for modeling. Immune markers included CD8B gene expression, CD8+ T cell infiltration estimated by CIBERSORT, and Immune Score estimated by ESTIMATE. Machine learning models, including Random Forest, Support Vector Machine, Deep Learning, and Elastic Net, were trained to classify immune-high versus immune-low tumors, followed by validation in an independent cohort of 13 patients. Results: Random Forest demonstrated strong internal performance (AUCs of 0.945, 0.993, and 0.974 for CD8B expression, CD8+ T cell infiltration, and Immune Score, respectively), but this dropped substantially in the held-out test partition and in an independent external validation cohort (*n* = 13, AUC 0.44–0.64), indicating that the internal metrics likely reflect overfitting at this sample size and should not be interpreted as evidence of generalizable performance. Additionally, we developed RadioImmune, a browser-based prototype research/demo tool that uses a logistic-regression approximation (not the Random Forest model above) for exploratory, real-time estimation of total immune score and CD8+ T cell infiltration status from radiomic inputs; its performance is not represented by the Random Forest AUCs reported above. Conclusions: These findings provide proof-of-concept that MRI-based radiomic features may serve as potential non-invasive biomarkers of the breast tumor immune microenvironment, pending validation in larger, independent cohorts.

## Simple Summary

The immune system plays an important role in breast cancer response to immunotherapy. Tumor-infiltrating lymphocytes (TILs) and CD8+ T cells provide important information about the tumor immune environment but are typically assessed using invasive tissue biopsies, which can be costly and burdensome for patients. This study explores whether breast MRI could provide a non-invasive approach to estimate tumor immune status. We analyzed MRI and gene-expression data from breast cancer patients and identified 18 radiomic features associated with immune activity. Machine-learning models were developed to predict immune status from these imaging features. The models showed strong performance in the initial cohort but more modest performance in an independent cohort, highlighting the need for validation in larger patient populations. We also developed Radioimmune, a browser-based research tool for estimating tumor immune status from MRI-derived radiomic features. Overall, these findings provide preliminary evidence that MRI-based radiomics may offer a non-invasive approach for assessing the breast tumor immune microenvironment and support further investigation of imaging-based biomarkers for immunotherapy research.

## 1. Introduction

Breast cancer remains the most diagnosed malignancy in women globally and a leading cause of cancer mortality [1]. The emergence of immune checkpoint inhibitors (ICIs), particularly anti-PD-1 and anti-PD-L1 agents, has meaningfully expanded the treatment landscape, especially for triple-negative and HER2-positive subtypes. Landmark trials including IMpassion130 and KEYNOTE-355 established pembrolizumab and atezolizumab in combination with chemotherapy as standard-of-care options for eligible patients with metastatic TNBC [2,3]. However, durable responses remain restricted to a subset of patients, and the molecular heterogeneity of breast cancer means that a single immunotherapeutic approach cannot uniformly benefit all tumor subtypes [4]. The identification of reliable, accessible biomarkers capable of predicting immune responsiveness prior to treatment initiation is therefore an urgent clinical priority.

The tumor immune microenvironment (TIME) is a critical regulator of immunotherapy efficacy. Tumors with abundant CD8+ cytotoxic T cell infiltration, classified as “immune-hot” or “immune -cold”, respond more favorably to checkpoint blockade than immune-excluded or immune-desert tumors [5,6]. CD8B gene expression serves as a specific transcriptomic marker of CD8+ T cell activity within tumors, while CIBERSORT (version: CIBERSORTx)-based deconvolution of bulk RNA-seq data provides quantitative estimates of CD8+ T cell fractional abundance [7]. Composite immune scores integrating multiple cell populations carry independent prognostic significance in breast cancer [8]. However, all these immune metrics are currently derived from post-surgical or biopsy-acquired tissue, making them subject to sampling bias, temporal delay, and the inherent invasiveness of tissue acquisition [9]. A non-invasive, pre-treatment method to assess tumor immune status would substantially improve the timeliness and accessibility of immunotherapy patient selection.

Radiomics addresses this gap by extracting hundreds of quantitative features capturing shape, intensity distribution, and spatial texture heterogeneity from standard medical imaging [10]. Because radiological phenotypes reflect not only intrinsic tumor cell biology but also the composition of the surrounding microenvironment, radiomic features have been proposed as macroscopic imaging correlates of underlying molecular and immunological processes [11,12]. The emerging field of radiogenomics formalizes this relationship by seeking systematic associations between imaging phenotypes and genomic or transcriptomic profiles [13]. Several recent studies demonstrate that MRI-derived radiomic features can predict immune infiltration status in breast cancer. Jeon et al. showed that DCE-MRI-based radiomic models could predict CD8+ T cell immunophenotypes in 182 breast cancer patients, with AUCs exceeding 0.80 in validation cohorts [14]. Qian et al. constructed MRI-based radiomic classifiers for prognosis-related immune cell infiltration using CIBERSORT deconvolution of TCGA-BRCA data linked to TCIA imaging, a framework closely analogous to the present study [15]. Han et al. demonstrated that an MRI-derived radiomic signature could estimate TME phenotypes defined by CD8+ infiltration status and achieved an AUC of 0.784 for predicting anti-PD-1/PD-L1 immunotherapy response in an independent immunotherapy-treated cohort [16].

The present study addresses questions about biologically grounded feature selection, model interpretability, and external generalizability by employing a set of 18 radiomic features pre-selected based on established literature associations with immune activity, and by evaluating performance across three complementary immune markers. This work represents a preliminary pilot analysis conducted on 38 patients available in the TCGA-BRCA/TCIA dataset; a fully powered analysis incorporating the complete cohort is planned as a subsequent study. The specific objectives were (1) to characterize the correlation structure between radiomic features and immune gene expression signatures; (2) to develop and compare four machine learning models for predicting immune-high versus immune-low tumor status; (3) to perform preliminary external validation of a four-model ensemble; and (4) to develop RadioImmune, a browser-based prototype research/demo tool operationalizing the trained model for exploratory, non-clinical use.

## 2. Materials and Methods

### 2.1. Study Design and Patient Cohorts

This was a retrospective, exploratory radiogenomic analysis. Imaging data and matched clinical information were obtained from TCIA, and gene expression data were obtained from TCGA-BRCA [17,18]. Two independent cohorts were defined: a discovery cohort of 38 patients with matched preoperative MRI and transcriptomic profiles, and an independent external validation cohort of 13 patients withheld from all model development steps. The 13-patient validation cohort was obtained from the QIN-BREAST-02 collection (The Cancer Imaging Archive; DOI: https://doi.org/10.7937/TCIA.2019.4cfm06rr), a distinct TCIA imaging collection independent of the source collection used for the 38-patient discovery cohort, and is therefore appropriately described as an external validation cohort rather than a held-out partition of the same collection. Cohort membership was determined solely by availability of complete paired imaging and molecular data. All data were de-identified and publicly accessible; no institutional review board approval was required. For clarity, three distinct cohort-related terms are used consistently throughout this manuscript: the “discovery cohort” refers to all 38 patients used for feature selection and model development; “training” and “test” refer to the 80/20 split (32 and 6 patients, respectively) used for internal evaluation within the discovery cohort; and the “external validation cohort” refers exclusively to the independent 13-patient QIN-BREAST-02 cohort, which was not involved in feature selection or model development in any capacity.

The discovery cohort comprised 38 unique patients (one sample identifier, TCGA-AR-A24M, had been inadvertently duplicated in an earlier data-consolidation step and is counted once here) across Luminal A (*n* = 26), Luminal B (*n* = 3), HER2-enriched (*n* = 4), and Basal-like (*n* = 4) subtypes, with one additional patient (TCGA-AR-A24S) of unrecorded/unavailable PAM50 subtype, and disease stages from Stage I to Stage IIIC. Treatments included anthracycline- and taxane-based chemotherapy, radiation therapy, hormone therapy, and, in a subset, trastuzumab or bevacizumab. Overall survival ranged from approximately 4 to 120 months. Full per-patient clinical and demographic characteristics are provided in Table S1.

### 2.2. MRI Acquisition and Preprocessing

Imaging data were downloaded from TCIA in DICOM format and loaded into 3D Slicer (version 5.10.0; www.slicer.org). Images were visually inspected slice-by-slice to confirm correct orientation, anatomical completeness, and absence of acquisition artifacts. No additional preprocessing steps (e.g., intensity normalization or N4 bias field correction) were applied beyond the resampling performed during feature extraction.

### 2.3. Tumor Segmentation

Manual tumor segmentation was performed using the Segment Editor module within 3D Slicer. The primary breast tumor was delineated slice-by-slice on the MRI series most suitable for volumetric delineation. The resulting three-dimensional tumor mask was saved as the region of interest (ROI) for all subsequent radiomic analyses. All segmentations were performed by a single operator with experience in breast tumor imaging; inter-observer variability assessment is planned for the expanded 139-patient analysis.

### 2.4. Radiomic Feature Extraction

The SlicerRadiomics extension (PyRadiomics, version: 5.10.0; https://pyradiomics.readthedocs.io) was applied to each patient’s tumor ROI [19]. Standardized extraction parameters were applied: voxel resampling to an isotropic resolution of 1 × 1 × 1 mm and gray-level discretization using a fixed bin count of 64. Extraction was performed on original (unfiltered) image data, yielding 107 original radiomic features per patient. This decision minimized feature redundancy and focused the analysis on the most directly interpretable feature classes.

### 2.5. Radiomic Feature Selection

From the 107 extracted original features, 18 biologically motivated features were selected using a dual-criterion strategy that combined Spearman correlation analysis against immune gene signatures with a systematic review of the radiomics-immunology literature, as detailed in Section 3.1 (Table 1). Two additional literature-informed, exploratory composite features were derived from primary extracted features: EdgeSharpness (Sphericity/VoxelVolume ratio, integrating tumor shape regularity and size as a proxy for margin morphology) and an AUC-inspired perfusion surrogate (First-Order Entropy × Intensity Range/100, integrating image heterogeneity and intensity spread to approximate contrast enhancement-related vascularity). They were evaluated as exploratory measures intended to capture immune-relevant imaging phenotypes not available as native PyRadiomics outputs.

The dual-criterion selection process described above initially yielded 20 candidate radiomic features. Two of these twenty were duplicate exports of the same underlying computation under different column names: first-order entropy and GLSZM zone entropy were each written out twice by the extraction pipeline under two naming variants (entropy/Entropy and Zone_Entropy/ZoneEntropy). These two duplicate-named columns were removed on that basis alone; a naming/redundancy criterion, not a statistical or performance-based exclusion criterion, leaving eighteen distinct radiomic features for model development, consistent with Table 1.

To assess the sensitivity of this feature-selection step to the train/test partition, we repeated this correlation analysis using only the 32-patient training set. The resulting correlation matrix was highly consistent with the one computed on the full 38-patient discovery cohort (Pearson r = 0.97; mean absolute difference per cell = 0.04), with 18 of the 20 most strongly associated features identical between the two analyses, indicating that feature selection is not meaningfully sensitive to whether the held-out test patients are included (Supplementary Figure S7).

### 2.6. Immune Marker Quantification

Three complementary immune markers were used as prediction targets. CD8B gene expression: RNA-seq-derived CD8B expression values obtained from TCGA-BRCA, used as a continuous measure of CD8+ T cell-specific transcriptional activity. CD8+ T cell infiltration (CIBERSORT): Deconvolution of bulk RNA-seq data to estimate fractional abundance of 22 immune cell populations, using CIBERSORTx (https://cibersortx.stanford.edu) run in relative mode against the LM22 reference signature matrix (22 immune cell subsets); only samples with a statistically significant deconvolution result (*p* < 0.05) were retained [21]. Total Immune Score: A global immune score integrating multiple immune cell populations, calculated to capture overall immune activity beyond the CD8+ compartment, analogous to the Immunoscore framework [8]. ESTIMATE scores were computed using the MD Anderson ESTIMATE web tool (bioinformatics.mdanderson.org/estimate), Disease-Centric Results, filtered to the Breast Invasive Carcinoma (BRCA) dataset using the RNA-Seq-V2 platform type.

### 2.7. Spearman Correlation Analysis

Spearman rank correlation coefficients (ρ) were calculated between the 18 radiomic features and 17 immune gene expression signatures representing major immune effector programs: CD8+ T cells, B cells, regulatory T cells (Treg), follicular helper T cells (Tfh), NK cells, macrophages, cytolytic activity (CYT), Type I and II interferon signaling, MHC Class I and II antigen presentation, immune checkpoints, and co-stimulatory molecules. Hierarchical clustering was applied to the resulting correlation matrix to identify coherent patterns of radiomic–immune association. Pairwise Spearman correlation analysis was also performed among the 17 immune signatures. All analyses were performed in R using the stats package; heatmaps were generated using the pheatmap package and annotated using ComplexHeatmap [22].

### 2.8. Machine Learning Model Development

Outcome binarization and classification evaluation: Regression models were trained on the continuous immune values using all 38 patients in the discovery cohort, per the 80/20 and 65/35 splits described below; the middle tertile was not removed from model training at any stage because the regression models are designed to predict continuous immune values across the full outcome range, and restricting training to only the extreme tertiles would discard information and artificially truncate that continuous distribution; tertile-based exclusion of the middle group was applied only later, and specifically, when developing the binary High/Low RadioImmune classifier described in Section 2.10, whose intended output requires sharply separated High versus Low classes. Tertile-based labels “High” (top tertile) or “Low” (bottom tertile) of measured immune values, with the middle tertile designated “Intermediate”, were applied only downstream of training, to the continuous actual values and to the continuous model predictions alike, in order to compute the classification metrics reported (AUC, accuracy, sensitivity). This tertile-based dichotomization follows extreme-contrast approaches used to define classification thresholds in prior radiomic immunophenotyping studies [14,15]. The resulting number of patients in each of the Low, Intermediate, and High categories for every immune target is reported in Table S3.

Model training: Four machine learning classifiers were trained independently using R (version: 4.5.3) and the caret package [23]: Random Forest (RF; 500 trees with bootstrapped feature sampling); Support Vector Machine (SVM; radial basis function kernel); Deep Learning (DL; single-hidden-layer feed-forward neural network implemented via the nnet package in R, with the number of hidden units and weight decay regularization tuned via the repeated 5-fold cross-validation grid search described above); Elastic Net (EN; L1 + L2 regularized regression, α = 0.5). Hyperparameters for each model were tuned internally using repeated 5-fold cross-validation (5 folds, 10 repeats) within the training partition, replacing an earlier leave-one-out cross-validation approach; this both stabilizes hyperparameter selection and provides a distribution of within-training-fold performance estimates in addition to the single held-out test-set evaluation. An 80/20 training–test split (*n* = 32 train, *n* = 6 test for CD8+ T cells and Immune Score; CD8B split to be confirmed against the regenerated *n* = 38 output) was applied as the primary evaluation framework. All three targets had complete data for all 38 patients; the 1-patient difference in exact partition size arises because caret’s create DataPartition stratifies continuous outcomes by internally binning them into quantile groups to preserve the overall distribution shape between train and test, so the closest achievable split to the requested 80/20 ratio can differ by a single patient depending on each target’s specific value distribution, even at identical *n* and *p*. This is expected behavior of stratified partitioning for continuous outcomes, not a missing-data artifact. A secondary 65/35 split (*n* = 25 train, *n* = 13 test, recalculated proportionally for *n* = 38; to be confirmed against regenerated output) was applied to assess sensitivity to partitioning. Model performance was evaluated using Pearson correlation (r) computed separately for the training and test partitions rather than pooled—between predicted and actual continuous immune values, AUC, accuracy, and sensitivity, computed using the pROC (version: 4.5.3) package [24]. Visualizations were generated using ggplot2 [25].

Classification thresholds: Optimal classification cutoffs were determined for each model via ROC curve analysis, selecting the probability score that maximized the Youden index (sensitivity + specificity − 1). Target-specific thresholds were applied (e.g., 0.58 for CD8B, 0.62 for CD8+ T cells, 0.50 for Immune Score).

### 2.9. Ensemble Validation

For the 13-patient validation cohort, the four trained models were applied independently to radiomic features alone. Each model generated a probability score for “High” immune infiltration; these were averaged into a single ensemble score per patient. Patients were classified as High (ensemble score ≥ 0.65), Low (≤0.35), or Intermediate. This ensemble approach reduces dependence on any single algorithm and provides more stable predictions in small external cohorts.

### 2.10. Prototype Research/Demo Tool Development

RadioImmune was implemented as a standalone HTML application accessible in any web browser. A Random Forest model (500 trees) was first trained in R on the same standardized 18-feature radiomic matrix used for the primary analysis (Table 1), from the 38-patient discovery cohort. The RadioImmune interface itself accepts 19 input fields because EdgeSharpness—1 of the 18 model features—is not entered directly; instead, the user supplies its two components, Sphericity and VoxelVolume, from which EdgeSharpness is computed internally before scoring. The model always operates on 18 features; 19 refers only to the number of raw fields presented in the user interface. A logistic regression model was subsequently fitted to the same training data; its intercept and coefficient vector, along with training-set feature means and standard deviations, were extracted from R and embedded in the application for real-time z-score normalization and inference. The tool accepts 19 radiomic feature values, computes a predicted probability of CD8+ T cell High infiltration status, and returns a classification of High (≥0.50), Intermediate, or Low, along with a feature importance visualization. No server, internet connection, or programming knowledge is required after initial page load.

RadioImmune’s real-time predictions are generated by a logistic regression model rather than the Random Forest classifier reported in Results. Because Random Forest cannot run natively in a static web browser, a logistic regression was separately fit on the same standardized training data to enable fully client-side inference: its intercept, coefficients, and training-set feature means and standard deviations were hard-coded into the HTML/JavaScript application for z-score normalization and logistic scoring. This model approximates rather than reproduces the Random Forest’s decision function, so its predictions are not guaranteed to match the Random Forest’s for every input.

### 2.11. Statistical Analysis

All statistical analyses were performed in R (https://www.r-project.org). Spearman correlation coefficients and two-sided *p*-values were computed using the R stats package. ROC curves and AUC values were computed using the pROC package [24]. Hierarchical clustering and heatmap visualizations were generated using the pheatmap package. Probability density estimation, bar charts, and scatter plots were produced using ggplot2 [25]. Statistical significance was defined at *p* < 0.05 throughout. The 95% confidence intervals for AUC values were computed using DeLong’s method as implemented in the pROC package, and are reported for each model and target in the performance tables in Section 3.

## 3. Results

### 3.1. Cohort Characteristics and Radiomic Feature and Genomic Feature Set

Constructing a paired imaging–genomic dataset required cross-referencing two independent repositories, as no single resource provides simultaneously accessible MRI images and matched transcriptomic data for breast cancer patients. The Cancer Genome Atlas Breast Cancer (TCGA-BRCA) collection provides molecular and clinical data for over 1000 patients, while the Cancer Imaging Archive (TCIA) independently hosts preoperative MRI images for a subset of these individuals. To bridge these resources, TCIA patient identifiers with confirmed MRI availability (*n* = 139) were used to retrieve matched RNA-seq HTSeq-FPKM expression data from the GDC Data Portal. After UUID-to-sample-ID resolution, files were consolidated into a single expression matrix and submitted to CIBERSORTx for immune cell deconvolution. Of the 139 TCIA-BRCA patients, 38 had both complete high-quality MRI segmentation and confirmed immune marker data, forming the final analysis cohort.

The discovery cohort comprised 38 unique patients (one sample identifier, TCGA-AR-A24M, was inadvertently duplicated in an earlier data-consolidation step and is counted once here) spanning four molecular subtypes: Luminal A (*n* = 26, 68.4%), Luminal B (*n* = 3, 7.9%), HER2-enriched (*n* = 4, 10.5%), and Basal-like (*n* = 4, 10.5%), with 1 additional patient (TCGA-AR-A24S) of unrecorded/unavailable PAM50 subtype, and disease stages ranging from Stage I to Stage IIIC. Treatments included anthracycline- and taxane-based chemotherapy, radiation therapy, hormone therapy (aromatase inhibitors and tamoxifen), and targeted agents (trastuzumab, bevacizumab). Overall survival ranged from 4.4 to 120.3 months, reflecting a broad spectrum of disease trajectories consistent with the clinical heterogeneity of the TCGA-BRCA cohort.

Radiomic features were extracted from manually delineated tumor ROIs in 3D Slicer using the SlicerRadiomics extension (PyRadiomics), with standardized isotropic resampling (1 × 1 × 1 mm) and fixed bin discretization (64 bins), consistent with the Image Biomarker Standardization Initiative (IBSI) framework [13]. From the full set of 107 original unfiltered radiomic features, 18 biologically motivated features were selected using the following dual-criterion strategy: features were first shortlisted based on Spearman correlation heatmap analysis against 17 immune gene signatures, then cross-referenced against published radiomic-immunology literature to confirm biological plausibility [14,15,16,20]. The final feature set spans six extraction categories—morphological shape, first-order intensity statistics, GLCM, GLRLM, GLSZM, and GLDM—and is summarized in Table 2. Two features were manually derived: EdgeSharpness (Sphericity ÷ VoxelVolume), as a surrogate for tumor margin irregularity, and AUC (Entropy × Range ÷ 100), as a proxy for intratumoral perfusion heterogeneity in the absence of true DCE-MRI pharmacokinetic parameters.

### 3.2. Spearman Correlation Between Radiomic Features and Immune Gene Signatures

To identify radiomic features with biologically meaningful associations with tumor immune activity, Spearman correlations were computed between all 107 extracted radiomic features and 17 immune gene expression signatures spanning cytotoxic, adaptive, innate, and immunosuppressive immune axes. This unsupervised heatmap analysis served as the data-driven first step in feature selection, enabling identification of features with coherent and reproducible correlation patterns across immune signatures before any predictive modeling was performed.

Hierarchical clustering of the full 107-feature correlation heatmap (Figure 1A) revealed that texture heterogeneity features—including first-order entropy, GLCM contrast, GLSZM zone entropy, and GLRLM run entropy—consistently co-clustered with cytotoxic immune signatures (CD8_T, CYT, IFN_Type2, MHC_ClassI), showing positive Spearman correlations across this immune axis. Morphological shape features (Sphericity, SurfaceVolumeRatio) exhibited weaker and more variable associations, while the AUC perfusion proxy showed limited correlation overall, consistent with the absence of true DCE-MRI dynamic contrast data in this cohort.

In the focused heatmap restricted to the final 18 selected features (Figure 1B), GLCM contrast and first-order entropy emerged as the most consistent positive correlates of cytotoxic immune activity, while DependenceNonUniformity and SizeZoneNonUniformity showed moderate associations with immune infiltration signatures. The radiomic feature-to-feature correlation matrix (Figure 1D) confirmed low inter-feature collinearity within the selected set, supporting their combined use as independent model inputs. Pairwise correlation analysis of the 17 immune signatures (Figure 1C) identified two co-expression modules: a cytotoxic/adaptive immunity cluster comprising CD8_T, CYT, IFN_Type2, MHC_ClassI, MHC_ClassII, Checkpoints, and Co_stimulation, and an innate/immunosuppressive cluster comprising Macrophages, Treg, and B_Cells. This modular structure confirmed that the three machine learning prediction targets—CD8B gene expression, CD8+ T cell infiltration (CIBERSORT), and Total Immune Score (ESTIMATE)—represent biologically coherent and complementary dimensions of the tumor immune microenvironment.

### 3.3. Machine Learning Classifier Performance

To determine whether MRI-derived radiomic features can non-invasively predict the immune composition of the tumor microenvironment, four machine learning algorithms—Random Forest (RF), Support Vector Machine (SVM), Deep Learning (DL), and Elastic Net (EN)—were independently trained to classify patients as immune-high or immune-low for each of the three immune targets. An 80/20 training–test split (*n* = 32 train, *n* = 6 test for CD8+ T cells and Immune Score; CD8B to be confirmed) was used as the primary evaluation framework. The 95% confidence intervals for all reported AUC values are provided alongside each model’s performance metrics in Figure 2C (CD8+ T cells) and Figure 3C (Immune Score).

#### 3.3.1. CD8+ T Cell Infiltration (CIBERSORT)

Predicting CIBERSORT-estimated CD8+ T cell fractions is the most direct test of whether tumor texture encodes cytotoxic immune infiltration, as these values reflect the actual immune cell composition deconvolved from bulk transcriptomic data. Classification thresholds were set at High ≥ 0.10 and Low ≤ 0.05 by tertile-based partitioning (Figure 2A). Random Forest achieved the strongest performance for this target (r = 0.949, *p* = 3.65 × 10^−20^, AUC = 0.993, accuracy = 0.949, sensitivity = 0.905), demonstrating near-perfect discrimination between immune-high and immune-low patients from radiomic features alone (Figure 2B,C). Corresponding full-cohort actual-versus-predicted correlations for the remaining three models are provided in Figure S1, and train/test split results under the 80/20 and 65/35 partitioning schemes are provided in Figures S2 and S3, respectively. The corresponding train/test split scatter plots are also shown directly in Figure 2D. SVM performed second (r = 0.715, AUC = 0.909, accuracy = 0.821, sensitivity = 0.810), confirming that the radiomic signal is robust across different model families. Deep Learning (r = 0.483, AUC = 0.729) and Elastic Net (r = 0.446, AUC = 0.679) showed lower but statistically significant performance, likely reflecting limited training sample size relative to model complexity. Actual CD8+ T cell scores were significantly higher in patients classified as High compared to Low (*p* = 2.1 × 10^−6^, Wilcoxon rank-sum test; Figure 2E). Patient-level predicted probabilities showed clear stratification into Low and High classes across the ranked heatmap (Figure 2F) and probability heatmap (Figure 2G).

#### 3.3.2. Total Immune Score (ESTIMATE)

The ESTIMATE-derived Total Immune Score integrates the fractional abundances of multiple immune cell populations—including CD8+ T cells, CD4+ memory T cells, regulatory T cells, NK cells, B cells, and macrophages—into a composite measure of overall immune infiltration. Predicting this composite target tests whether radiomic features capture broader immune activity beyond the CD8+ cytotoxic axis. Classification thresholds were High ≥ 1303.09 and Low ≤ 603.42 (Figure 3A). Random Forest again achieved the strongest performance (r = 0.960, *p* = 5.78 × 10^−22^, AUC = 0.974, accuracy = 0.846, sensitivity = 0.700; Figure 3B,C), followed by SVM (r = 0.796, AUC = 0.889, accuracy = 0.718, sensitivity = 0.800) and Elastic Net (r = 0.499, AUC = 0.713, accuracy = 0.692, sensitivity = 0.700), Figure 3B,C). (Random Forest, SVM, Elastic Net, Deep Learning) were trained on the continuous immune values using all 38 patients in the discovery cohort, per the 80/20 and 65/35 splits described in Methods. The middle tertile was not excluded from regression model training at any stage. This is because the regression models are designed to predict continuous immune values across the full outcome range; restricting training to only the extreme (top/bottom) tertiles would discard information and artificially truncate that continuous distribution. Corresponding full-cohort actual-versus-predicted correlations for the remaining three models are provided in Figure S4, and train/test split results under the 80/20 and 65/35 partitioning schemes are provided in Figures S5 and S6, respectively. The corresponding train/test split scatter plots are also shown directly in Figure 3D. Deep Learning showed convergence failure for this target (r = 0.162, *p* = 0.325, AUC = 0.572, sensitivity = 0.200), with predicted values concentrated near 1400, indicating that the Deep Learning architecture was underpowered for a continuous composite score at this sample size. Actual Immune Scores were significantly higher in High-classified patients (*p* = 1.6 × 10^−5^, Wilcoxon rank-sum test; Figure 3E). The ranked probability heatmap (Figure 3F) and per-patient probability display (Figure 3G) confirmed coherent stratification across the 38-patient cohort. “High” (top tertile), “Low” (bottom tertile), and “Intermediate” (middle tertile) were applied only downstream of training to both the continuous actual values and the continuous model predictions to compute the classification metrics reported (AUC, accuracy, sensitivity). This tertile-based dichotomization follows extreme-contrast approaches used to define classification thresholds in prior radiomic immunophenotyping studies.

### 3.4. Ensemble Validation in the Independent 13-Patient Cohort

Internal model performance, even on an external cohort, can overestimate true generalizability when the training and test sets are drawn from the same patient population and imaging protocol. To assess whether the radiomic–immune associations identified in the 38-patient cohort extend to unseen patients, the four trained models were applied to radiomic features from 13 patients completely withheld from all model development steps. Per-patient probability scores from the four models were averaged into a single ensemble score, and patients were classified as High (ensemble ≥ 0.65), Low (≤0.35), or Intermediate. The 13-patient validation cohort was obtained from the QIN-BREAST-02 collection (The Cancer Imaging Archive; DOI: https://doi.org/10.7937/TCIA.2019.4cfm06rr), a distinct TCIA imaging collection independent of the source collection used for the 38-patient discovery cohort, and is therefore appropriately described as an external validation cohort rather than a held-out partition of the same collection.

External validation AUCs ranged from 0.444 to 0.636 across the three immune targets. Full per-patient ensemble prediction results for this cohort are provided in Table S2 CD8B showed the most consistent generalization, with three of four models achieving AUCs above chance (RF = 0.619, Deep Learning = 0.615, Elastic Net = 0.636) and the most distributed class predictions across High, Intermediate, and Low groups (High = 6/13, 46%; Intermediate = 5/13, 38%; Low = 2/13, 15%; Figure 4B). For CD8+ T cell infiltration, performance was marginal (AUC range 0.473–0.540), with the majority of validation patients assigned to the Intermediate class (12/13, 92.3%), indicating that model confidence was insufficient to commit to binary classification in the external cohort. Total Immune Score showed mixed performance: Elastic Net (AUC = 0.583) and Deep Learning (AUC = 0.549) retained modest discriminative capacity, while Random Forest (AUC = 0.460) and SVM (AUC = 0.446) fell at or below chance level.

The gap between internal AUCs (0.679–0.993) and external validation AUCs (0.444–0.636) reflects the overfitting expected in small-sample radiomics studies and is consistent with the established literature on radiomics model generalizability [26]. CD8B outperformed CIBERSORT-derived fractions in external validation despite comparable internal AUCs, suggesting that single-gene transcriptomic endpoints may be more externally generalizable than algorithmically deconvolved cell fractions at this sample size. These findings underscore the need for expanded validation in the planned 130-patient TCGA-BRCA/TCIA cohort, which is expected to substantially improve both model stability and external performance, as demonstrated by Jeon et al. [14], who reported validation AUCs of 0.973–0.993 in a 182-patient DCE-MRI cohort.

### 3.5. Development of RadioImmune—Browser-Based Prototype Research/Demo Tool

Translating radiomic immune prediction into clinical practice requires that predictions be accessible without specialized software, statistical expertise, or server infrastructure. To address this, RadioImmune was developed as a standalone, browser-accessible prototype research/demo tool that enables real-time, exploratory CD8+ T cell infiltration classification from 19 radiomic feature values extracted from a patient’s preoperative breast MRI.

A Random Forest model (500 trees) was trained in R on the same standardized 18-feature radiomic matrix used for the primary analysis, from the 38-patient cohort. To enable browser-based inference without a server backend, a logistic regression model was subsequently fitted to the same training data, and its intercept and coefficient vector, along with training-set feature means and standard deviations, were embedded directly in the HTML application. This approach allows real-time z-score normalization of user-entered feature values and instantaneous logistic inference entirely within the browser.

The tool accepts 18 radiomic feature values as input (EdgeSharpness is computed internally from Sphericity and VoxelVolume), returns a predicted probability of High CD8+ T cell infiltration P(High), classifies the patient as High (*p* ≥ 0.50), Intermediate, or Low, and displays the Random Forest variable importance ranking for the submitted feature set (Figure 5A–C). Tertile-based exclusion of the middle group was applied only later, specifically when developing the binary High/Low RadioImmune classifier (Section 2.10), whose intended output requires sharply separated High versus Low classes for a meaningful binary decision boundary. A methodology panel explains the full computational pipeline from radiomic extraction to logistic inference (Figure 5D). RadioImmune is provided as Supplementary File S1, requires no installation or internet connection after initial page load, and will be updated with model coefficients from the expanded 130-patient analysis.

## 4. Discussion

This preliminary radiogenomic analysis provides proof-of-concept evidence that MRI-derived radiomic features capture quantitative imaging phenotypes associated with CD8+ T cell infiltration and overall immune activity in breast cancer. Using a matched dataset from TCIA and TCGA-BRCA, we demonstrate that texture and intensity heterogeneity features—particularly entropy, GLCM contrast, and zone entropy—are meaningfully correlated with immune gene expression signatures reflecting cytotoxic T cell activity, interferon signaling, and antigen presentation. These associations are biologically coherent: tumors with heterogeneous texture patterns on imaging tend to exhibit more complex, activated microenvironments consistent with immune infiltration and tissue remodeling [10,11].

Our machine learning findings are consistent with the radiogenomics literature. Jeon et al. [14] trained DCE-MRI radiomic models to predict CD8+ T cell immunophenotypes in 182 breast cancer patients and demonstrated that multi-phase radiomic signatures outperformed single-phase models, with AUC ranges comparable to our Random Forest results. Qian et al. [15], who used CIBERSORT-derived immune cell fractions as radiomic prediction targets in a TCIA-TCGA matched framework directly analogous to our approach, further support the validity of this analytical strategy. Han et al. [16] additionally demonstrated that MRI-derived radiomic signatures can predict immunotherapy response in anti-PD-1/PD-L1-treated cohorts, establishing the clinical relevance of the radiomic–immune axis. A key strength of the present study is the simultaneous evaluation of three complementary immune markers—transcriptomic gene expression (CD8B), cell-fraction deconvolution (CIBERSORT), and composite immune scoring—providing a more comprehensive characterization of the immune landscape than any single marker alone.

Several limitations require acknowledgment. First, the discovery cohort of 38 patients is modest, and the performance metrics reported here—while internally consistent—are derived from small held-out partitions (*n* = 6 for the 80/20 split) and cannot be interpreted as definitive estimates of clinical performance. The external validation results confirm this concern: ensemble AUCs of 0.54–0.67 in 13 independent patients reflect modest generalizability and underscore the need for substantially larger validation studies. The collapse of the CD8+ T cell classifier to the Intermediate category in the external cohort most likely reflects overfitting on the small discovery set, compounded by potential distributional differences between cohorts.

The gap between internal and external performance in this study is best understood as an expected consequence of the sample-size-to-model-complexity ratio rather than an isolated anomaly. With 38 discovery patients, 18 radiomic features, and five machine learning algorithms independently compared for each of three immune targets, the number of model parameters and pairwise comparisons is large relative to the number of independent observations, a setting in which even a held-out 20% test partition (*n* = 6) offers limited protection against overfitting because the same small pool of patients informs both feature selection and threshold determination upstream of the train/test split. Consistent with this, the higher-capacity, more flexible algorithms in our comparison—Random Forest and the Deep Learning model—showed the largest drop between internal and external AUC, while Elastic Net, which is more constrained by its L1/L2 regularization, degraded less. This pattern is consistent with the general expectation that more flexible models are more prone to fitting cohort-specific noise in small-sample settings. Taken together, the internal performance metrics reported here should be interpreted as optimistic, best-case estimates rather than expected real-world performance, and the external validation results in the 13-patient cohort—despite their own small size—provide a more realistic, if still preliminary, indication of generalizability.

Second, the MRI sequences used for feature extraction were not uniformly DCE-MRI sequences. Dynamic contrast-enhanced MRI has been preferred in prior breast radiomics studies because pharmacokinetic contrast enhancement properties directly relate to tumor vascularity and immune access [14,15]. The surrogate AUC feature approximates perfusion dynamics from static imaging, which is a recognized limitation; true DCE-derived perfusion metrics would strengthen biological grounding. Third, manual segmentation by a single operator introduces reproducibility risk; inter-observer agreement assessment is a prerequisite for clinical translation [26] and is planned for the expanded analysis. Beyond inter-observer agreement, feature-level robustness has not yet been assessed: texture features such as GLCM contrast and GLSZM zone entropy—identified here as the most immune-associated radiomic phenotypes—are generally more sensitive to small variations in segmentation boundaries than first-order or shape features, so a formal test–retest or boundary-perturbation analysis will be needed to establish how stable these specific associations are before they can be considered reliable imaging biomarkers. Fourth, equal feature weighting in the radiomic score represents a starting point; data-driven weighting via penalized regression or bootstrap importance scores should be incorporated in the expanded analysis.

Despite these limitations, the identification of texture-heterogeneity features as the most consistently immune-associated radiomic phenotypes supports a conceptual model in which high intratumoral spatial variability on MRI—reflecting a complex, activated microenvironment—is a macroscopic manifestation of immune infiltration [11,15]. Given the modest external validation performance observed here (ensemble AUCs of 0.44–0.67 in 13 independent patients), these associations should be regarded as hypothesis-generating rather than clinically actionable at this stage. If validated prospectively in substantially larger, independent cohorts, such imaging biomarkers might eventually help support pre-treatment immune stratification without biopsy, potentially assisting in the identification of patients with immune-hot tumors most likely to benefit from checkpoint inhibitor therapy [5,6]. The development of RadioImmune as a browser-accessible, zero-dependency prototype research/demo tool represents an important step toward removing the computational barriers that typically limit research uptake of radiomic machine learning models.

## 5. Conclusions

This preliminary radiogenomic analysis provides proof-of-concept evidence that quantitative MRI-derived radiomic features, particularly texture heterogeneity measures including entropy, GLCM contrast, and zone entropy, may serve as potential imaging signatures associated with immune gene expression and CD8+ T cell infiltration status in breast cancer, pending validation in larger, independent cohorts. A multi-model machine learning pipeline achieved strong discriminative performance for immune-high versus immune-low classification within the 38-patient discovery cohort, and an ensemble approach yielded moderate preliminary external validation signal in 13 independent patients. The development of RadioImmune—a standalone, browser-based prototype research/demo tool enabling real-time, exploratory radiomic immune prediction without computational expertise—represents a concrete translational output of this proof-of-concept analysis. The expanded analysis incorporating the full 139-patient cohort will test whether these radiomic–immune associations are sufficiently reproducible to support clinical translation as a non-invasive adjunct to immunotherapy patient selection.

## Figures and Tables

**Figure 1:**
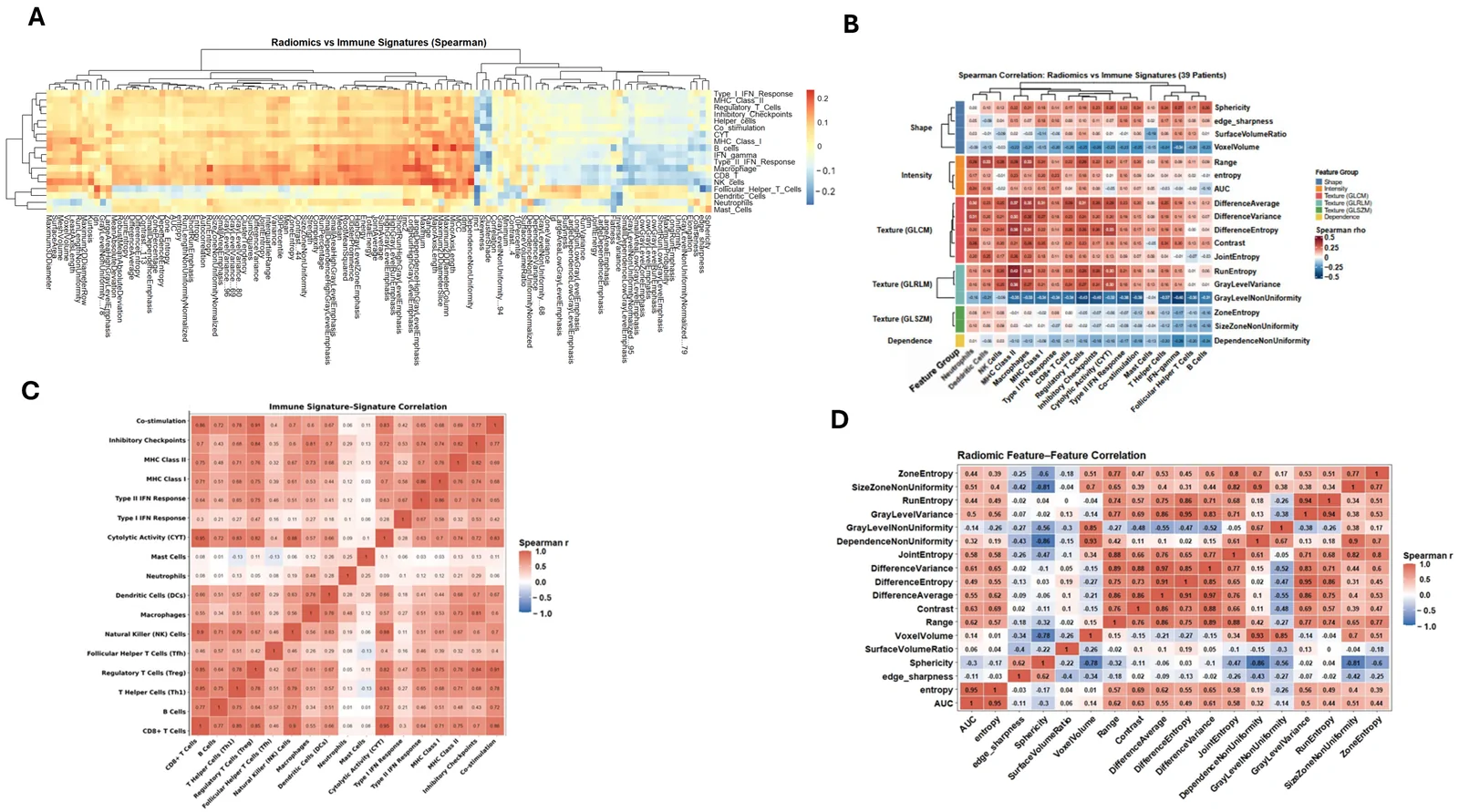
Radiomic and immune landscape of the 38-patient TCGA-BRCA cohort. (**A**) Spearman correlations between radiomic features and 17 immune gene signatures. (**B**) Correlations between selected radiomic features across six feature categories and immune signatures. (**C**) Pairwise correlations among the 17 immune signatures, showing clustered immune pathway relationships. (**D**) Correlation matrix of selected radiomic features illustrating inter-feature collinearity used to guide feature selection.

**Figure 2:**
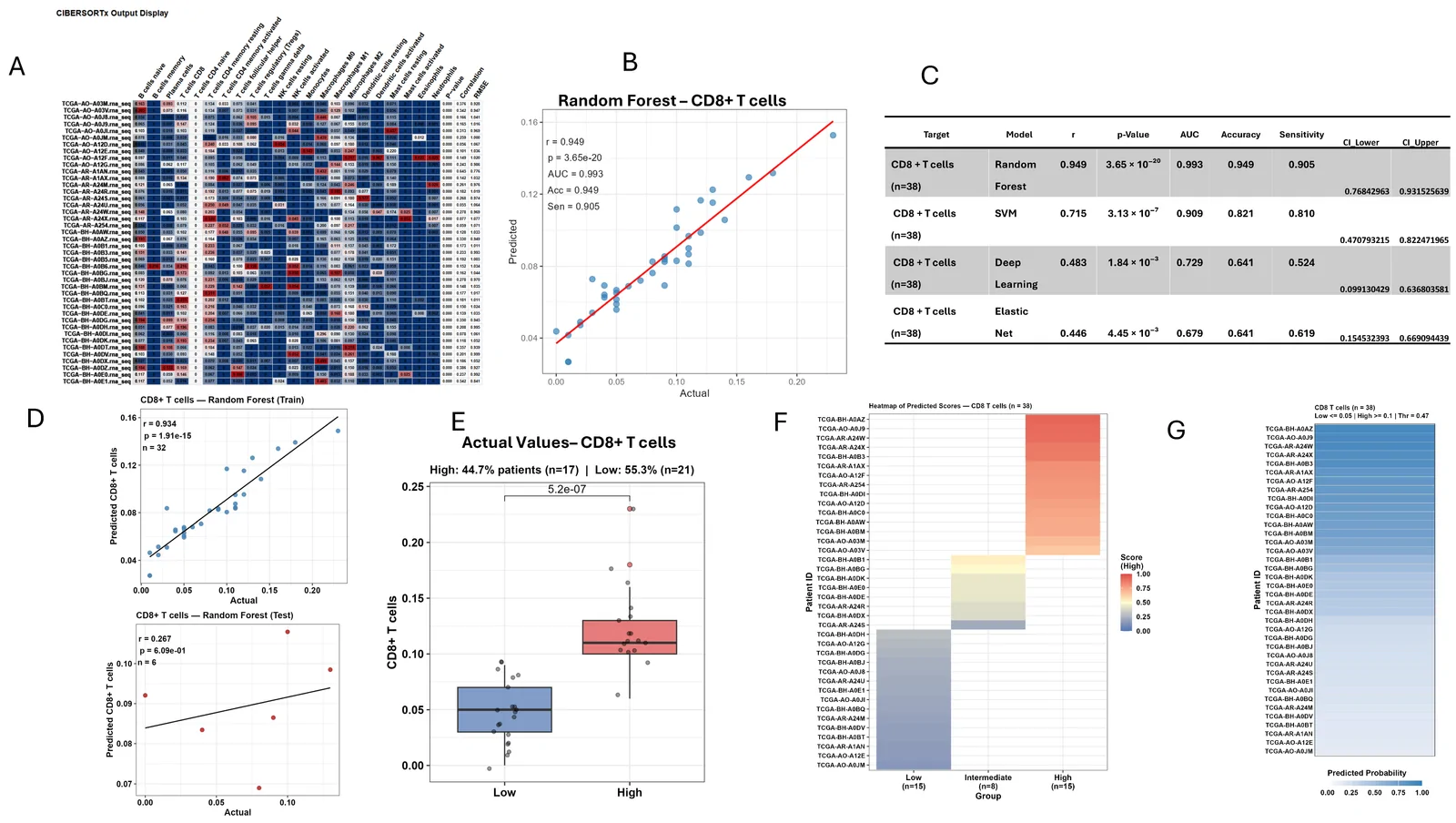
Radiomic prediction of CD8+ T cell infiltration in TCGA-BRCA. (**A**) CIBERSORT-derived immune cell fractions across 38 patients used to define the CD8+ T cell prediction target. (**B**) Actual versus predicted CD8+ T cell scores from the best-performing Random Forest model (r = 0.949, *p* = 3.65 × 10^−20^, AUC = 0.993, accuracy = 0.949, sensitivity = 0.905). Red line indicates perfect prediction. (**C**) Performance comparison of Random Forest, SVM, Deep Learning, and Elastic Net models. (**D**) Random Forest predictions after repeated 5-fold cross-validation, shown separately for training (*n* = 32; r = 0.934, *p* = 1.91 × 10^−15^) and test sets (*n* = 6; r = 0.267, *p* = 0.609). (**E**) Actual CD8+ T cell scores for Low (≤0.05) and High (≥0.1) groups (Wilcoxon *p* = 2.1 × 10^−6^). (**F**) Predicted CD8+ T cell scores stratified as Low, Intermediate, or High. (**G**) Predicted probability of the High CD8+ T cell infiltration class, sorted from low to high.

**Figure 3:**
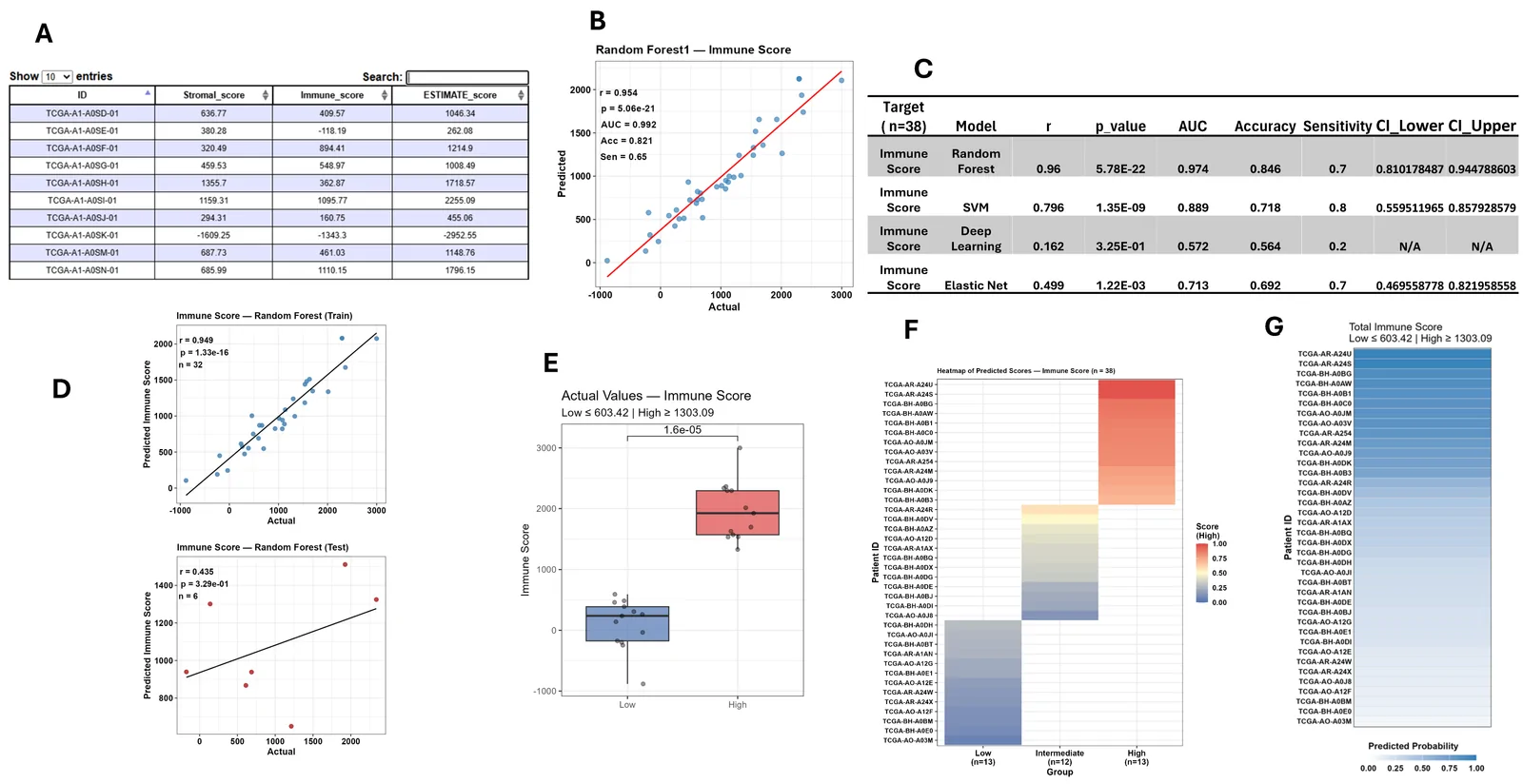
Radiomic prediction of total immune score in TCGA-BRCA. (**A**) ESTIMATE-derived Stromal, Immune, and ESTIMATE Scores used as prediction targets. (**B**) Actual versus predicted Immune Scores from the best-performing Random Forest model (r = 0.96, *p* = 5.78 × 10^−22^, AUC = 0.974, accuracy = 0.846, sensitivity = 0.70). Red line indicates perfect prediction. (**C**) Performance comparison of Random Forest, SVM, Deep Learning, and Elastic Net models. (**D**) Random Forest predictions after repeated 5-fold cross-validation, shown separately for training (*n* = 32; r = 0.949, *p* = 1.33 × 10^−16^) and test sets (*n* = 6; r = 0.435, *p* = 0.329). (**E**) Actual Immune Scores for Low (≤603.42) and High (≥1303.09) groups (Wilcoxon *p* = 1.6 × 10^−5^). (**F**) Predicted Immune Scores stratified as Low, Intermediate, or High. (**G**) Predicted probability of the High Immune Score class, sorted from low to high.

**Figure 4:**
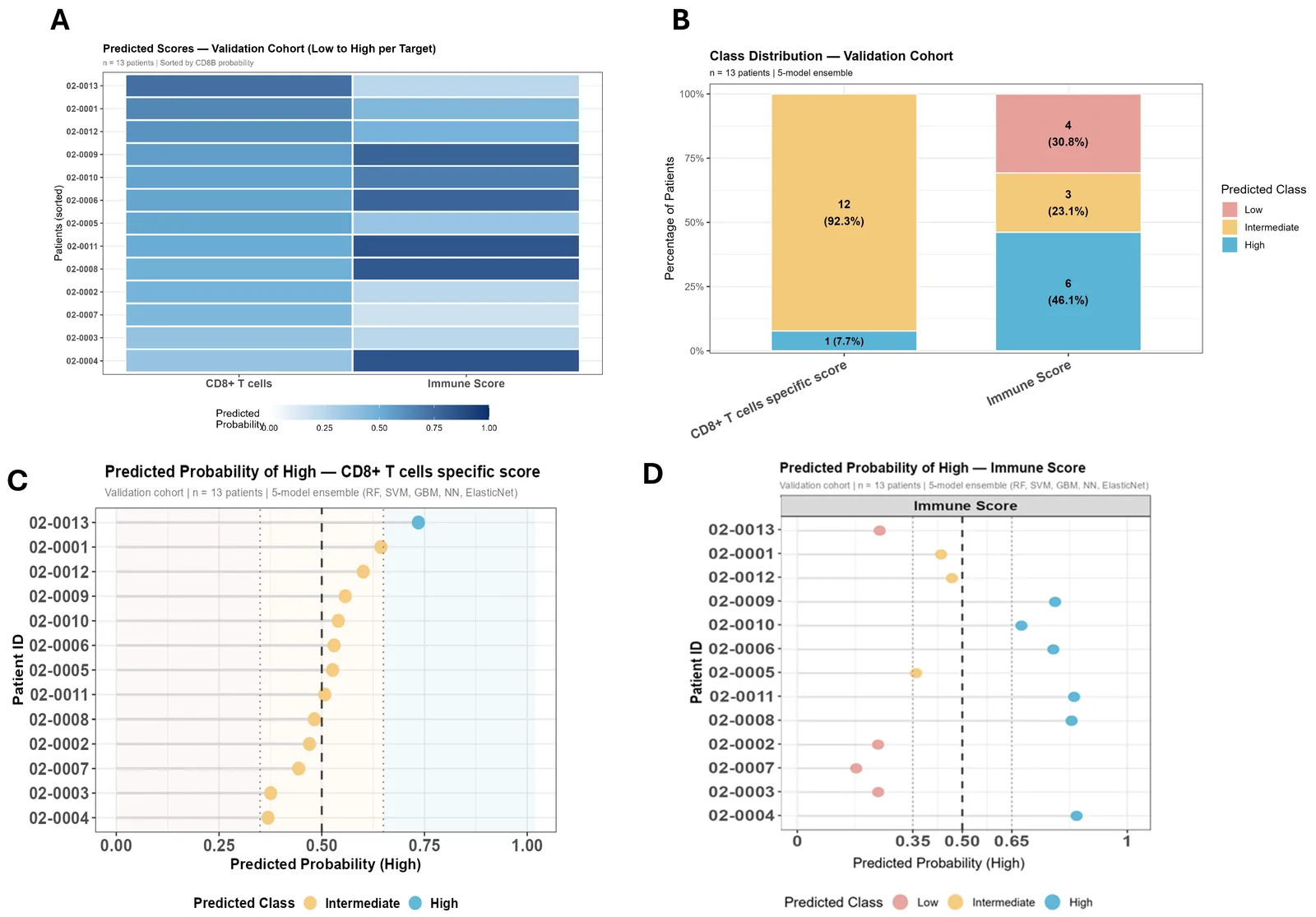
Ensemble model validation in an independent 13-patient cohort. Four trained models (Random Forest, SVM, Deep Learning, and Elastic Net) were applied to an independent validation cohort, and their predicted probabilities were averaged for ensemble classification. (**A**) Ensemble-predicted probabilities for CD8+ T cells and Immune Score, sorted by CD8+ T cell probability. (**B**) Distribution of Low, Intermediate, and High classifications for each immune target. (**C**) Per-patient ensemble probability of High CD8+ T cell infiltration, with the 0.50 classification threshold indicated. (**D**) Per-patient ensemble probability of High Immune Score, with the 0.50 threshold indicated.

**Figure 5:**
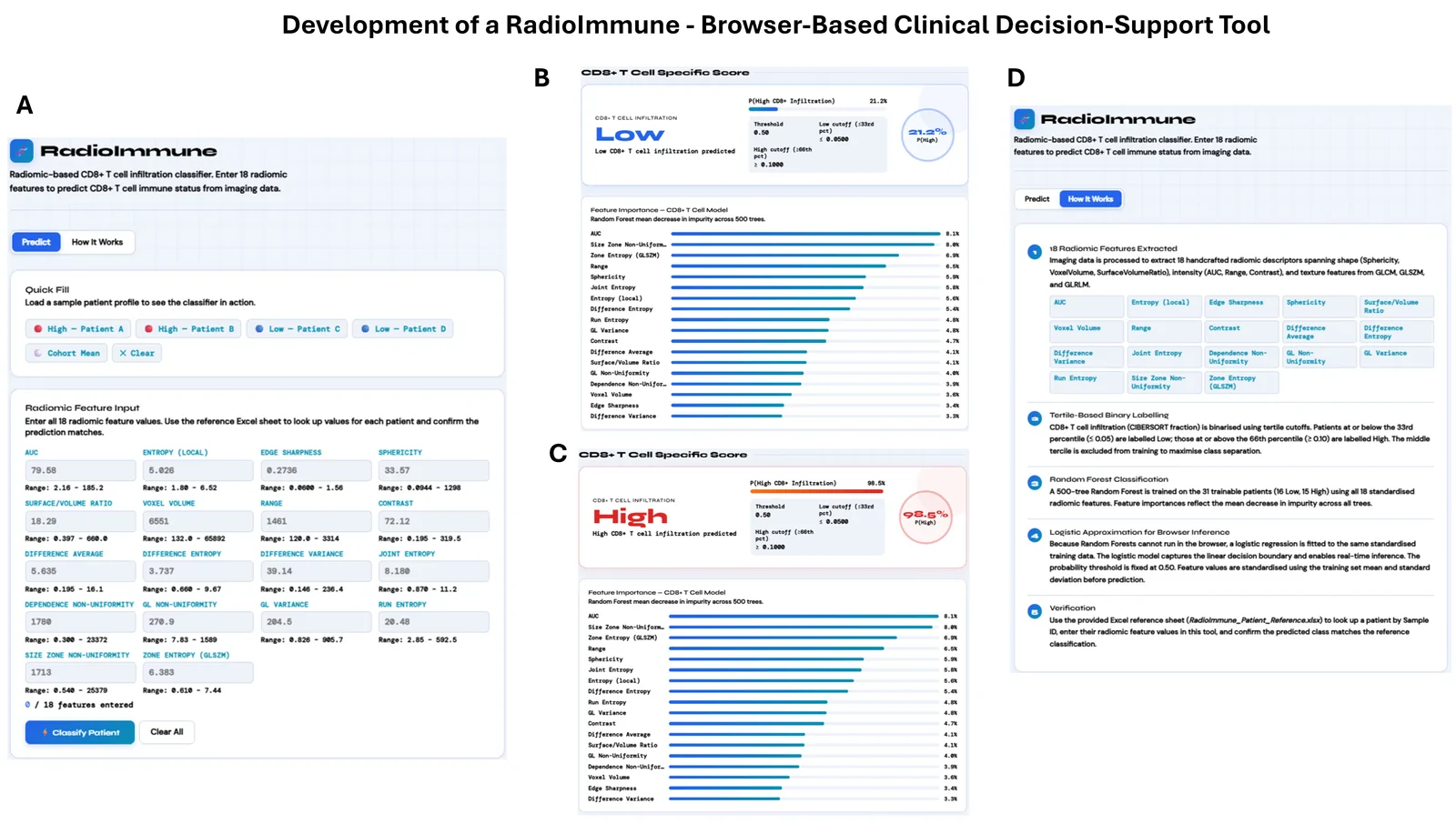
Development of the RadioImmune browser-based prototype research/demo tool. (**A**) RadioImmune interface for entering standardized MRI-derived radiomic features and generating CD8+ T cell infiltration predictions. (**B**) Example output showing Low CD8+ T cell infiltration (21.2% predicted probability), classification threshold, confidence interval, and Random Forest feature importance. (**C**) Example output showing High CD8+ T cell infiltration (98.8% predicted probability), classification threshold, confidence interval, and Random Forest feature importance. (**D**) Five-step RadioImmune workflow: radiomic feature extraction, tertile-based labeling, Random Forest training, browser-based inference, and reference-patient verification.

**Table 1:** Selected radiomic features and their biological rationale. Radiomic features used in model development are organized by extraction categories: morphological shape descriptors, first-order intensity statistics, and four texture matrix families (GLCM, GLRLM, GLSZM, GLDM). For each category, the specific features selected and their biological relevance to CD8+ T cell infiltration and tumor immune microenvironment are described.

Category	Full Name	Feature(s)	Biological Rationale	References
Shape	Morphological	Sphericity, SurfaceVolumeRatio, VoxelVolume, EdgeSharpness	Irregular tumor shape and high surface-to-volume ratio reflect infiltrative growth and stromal remodeling that limit CD8+ T cell access.	[14,15,20]
First-Order	Intensity Statistics	AUC, Entropy, Range	High entropy and wide intensity range indicate intratumoral metabolic heterogeneity linked to hypoxic niches and reduced immune infiltration.	[10,11]
GLCM	Gray Level Co-occurrence Matrix	Contrast, DifferenceAverage, DifferenceEntropy, DifferenceVariance, JointEntropy	Spatial intensity relationships encode local tissue texture; altered GLCM patterns reflect stromal remodeling associated with immune infiltration.	[14,16]
GLRLM	Gray Level Run Length Matrix	RunEntropy, GLNonUniformity, GLVariance	Non-uniform intensity run patterns indicate mixed cellularity and necrotic regions where immune infiltrates may be embedded.	[15,20]
GLSZM	Gray Level Size Zone Matrix	SizeZoneNonUniformity, ZoneEntropy	High zone entropy reflects heterogeneous microenvironments consistent with immune activation and T cell infiltration.	[14,20]
GLDM	Gray Level Dependence Matrix	DependenceNonUniformity	Regional variation in pixel intensity dependence mirrors heterogeneous cellular density correlating with differential immune cell accumulation.	[15,20]

**Table 2:** Immune gene signatures used for CIBERSORT-based immune deconvolution. Seventeen immune cell types and functional signatures were defined using curated gene sets applied to TCGA-BRCA RNA-seq data. Gene sets include lineage markers for adaptive and innate immune populations, functional activity signatures (cytolytic activity, IFN response), antigen presentation machinery (MHC Class I and II), and immune regulatory pathways (inhibitory checkpoints, co-stimulation).

Immune Gene Signature	Genes
CD8 + T Cells	*CD8A*, *CD8B*, *CD3D*, *CD3E*, *CD3G*
B Cells	*CD19*, *CD79A*, *CD79B*, *MS4A1* (*CD20*), *BANK1*
T Helper Cells (Th1)	*IFNG*, *TBX21* (*T-bet*), *STAT4*, *IL12RB2*
Regulatory T Cells (Treg)	*FOXP3*, *IL2RA (CD25)*, *CCR4*, *CTLA4*
Follicular Helper T Cells (Tfh)	*BCL6*, *CXCL13*, *ICOS*, *CXCR5*
Natural Killer (NK) Cells	*NCAM1 (CD56)*, *NCR1* (*p46*), *KLRB1*, *CD160*, *PRF1*
Macrophages	*CD68*, *CD163*, *CD86*, *MRC1*, *CSF1R*
Dendritic Cells (DCs)	*CD1C*, *CD1E*, *ITGAX* (*CD11c*), *CLEC4C*
Neutrophils	*CEACAM8*, *FCGR3B*, *CXCR1*, *CXCR2*
Mast Cells	*TPSAB1*, *TPSB2*, *CPA3*, *MS4A2*
Cytolytic Activity (CYT)	*GZMA*, *PRF1*
Type I IFN Response	*IFIT1*, *IFIT3*, *MX1*, *ISG15*, *OAS1*
Type II IFN Response	*IFNG*, *IRF1*, *STAT1*, *PSMB9*, *IDO1*
MHC Class I	*HLA-A*, *HLA-B*, *HLA-C*, *B2M*, *TAP1*, *TAP2*
MHC Class II	*HLA-DPA1*, *HLA-DPB1*, *HLA-DRA*, *HLA-DRB1*, *CD74*
Inhibitory Checkpoints	*PDCD1* (*PD-1*), *CD274* (*PD-L1*), *CTLA4*, *LAG3*, *HAVCR2* (*TIM-3*)
Co-stimulation	*CD28*, *CD40L*, *CD27*, *ICOS*, *TNFRSF4* (*OX40*)

## Data Availability

Imaging data for the 38-patient discovery cohort are publicly available from The Cancer Imaging Archive (TCIA; https://www.cancerimagingarchive.net). Imaging data for the 13-patient external validation cohort are publicly available from the QIN-BREAST-02 collection on TCIA (DOI: https://doi.org/10.7937/TCIA.2019.4cfm06rr). Transcriptomic data are publicly available from The Cancer Genome Atlas (TCGA; https://portal.gdc.cancer.gov). The RadioImmune tool is provided as Supplementary File S1.

## Supplementary Materials

The following supporting information can be downloaded at: https://www.mdpi.com/article/10.3390/cancers18193087/s1, Table S1: TCGA-BRCA training cohort patient characteristics; Table S2: Validation cohort ensemble prediction results for 13 withheld patients; Table S3: Number of patients classified as Low, Intermediate, and High for each immune target based on tertile thresholds; Figure S1: Correlation between actual and predicted immune scores—CD8+ T cell infiltration (CIBERSORT); Figure S2: Train versus test set predicted probabilities—CD8+ T cell infiltration (CIBERSORT); Figure S3: Train versus test set predicted values for CD8+ T cells across four machine learning models (Deep Learning, Elastic Net, Random Forest, SVM), secondary 65/35 split (*n* = 25 train, *n* = 14 test), assessing sensitivity-to-partition ratio; Figure S4: Correlation between actual and predicted immune scores—Total Immune Score (ESTIMATE). Figure S5: Train versus test set predicted probabilities—Total Immune Score (ESTIMATE). Figure S6: Train versus test set predicted values for Total Immune Score (ESTIMATE) across four machine learning models (Deep Learning, Elastic Net, Random Forest, SVM), secondary 65/35 split (*n* = 25 train, *n* = 14 test), assessing sensitivity to partition ratio. Figure S7: Feature-selection correlation stability at *n* = 32. File S1: RadioImmune tool.
