# Supplementary material for: Evaluation of MRI Radiomic Features as Surrogates of Tumor Immune Status in Breast Cancer: A Pilot Study of MRI-Derived Radiomic Features for Non-Invasive Immune Profiling in Breast Cancer
Source: Cancers (Basel). 2026 Sep 23;18(19):3087. doi: 10.3390/cancers18193087 (PMC13664318; doi:10.3390/cancers18193087)
Supplement: Supplementary file 1 [file cancers-18-03087-s001.zip › cancers-4555058-supplementary.pdf]

Supplementary Table S1. TCGA-BRCA training cohort patient characteristics.

Molecular subtype and pathological stage for the 39 patients comprising the model training cohort, sourced from TCGA-BRCA. Molecular subtypes were determined by PAM50 classification (LumA = Luminal A, LumB = Luminal B, HER2 = HER2-enriched, Basal = Basal-like).

TCGA Sample ID	Subtype	Stage
**TCGA-AO-A0J8**	LumA	IIA
**TCGA-AO-A0J9**	LumA	IIIC
**TCGA-AO-A0JI**	LumA	IIA
**TCGA-AO-A0JM**	LumB	IIB
**TCGA-AO-A03M**	LumA	I
**TCGA-AO-A03V**	LumA	I
**TCGA-AO-A12D**	HER2	IIA
**TCGA-AO-A12E**	LumA	IIB
**TCGA-AO-A12F**	Basal	IIA
**TCGA-AO-A12G**	LumA	IIA
**TCGA-AR-A24M**	LumA	IIA
**TCGA-AR-A1AN**	LumA	I
**TCGA-AR-A1AX**	LumA	IIIA
**TCGA-AR-A24R**	LumB	I
**TCGA-AR-A24S**	—	IIIA
**TCGA-AR-A24U**	HER2	IIA
**TCGA-AR-A24W**	LumA	IIB
**TCGA-AR-A24X**	LumA	IIA
**TCGA-AR-A254**	HER2	IIIA
**TCGA-BH-A0AW**	HER2	IIA
**TCGA-BH-A0AZ**	LumA	IIIA
**TCGA-BH-A0B1**	LumA	I
**TCGA-BH-A0B3**	Basal	IIB
**TCGA-BH-A0BG**	Basal	I
**TCGA-BH-A0BJ**	LumA	IIB
**TCGA-BH-A0BM**	LumA	IIB
**TCGA-BH-A0BQ**	LumA	I
**TCGA-BH-A0BT**	LumA	IIA
**TCGA-BH-A0C0**	LumB	IIA
**TCGA-BH-A0DE**	LumA	IIA
**TCGA-BH-A0DG**	LumA	IIA
**TCGA-BH-A0DH**	LumA	IIB
**TCGA-BH-A0DI**	LumA	IIB
**TCGA-BH-A0DK**	LumA	IIA
**TCGA-BH-A0DV**	LumA	IIIA
**TCGA-BH-A0DX**	LumA	I
**TCGA-BH-A0E0**	Basal	IIIC
**TCGA-BH-A0E1**	LumA	IIB

Supplementary Table S2. Validation cohort ensemble prediction results for 13 withheld patients.
Per-patient ensemble-predicted probabilities of High immune infiltration and corresponding class assignments (High, Intermediate, or Low) for CD8B gene expression, CD8+ T cell infiltration (CIBERSORT), and Total Immune Score (ESTIMATE) across 13 patients withheld from all model development steps. Ensemble scores represent the average predicted probability across four models (Random Forest, SVM, Deep Learning, Elastic Net). The final column indicates the number of individual models that independently predicted High (≥ 0.50) for each patient. Patients with ensemble scores ≥ 0.65 were classified as High, ≤ 0.35 as Low, and between 0.35 and 0.65 as Intermediate.

Patient ID	CD8B P(High)	CD8B Class	T Cells CD8 P(High)	T Cells CD8 Class	Immune Score P(High)	Immune Score Class	Models Agreeing High
**02-0001**	0.794	High	0.646	High	0.348	Low	3/4
**02-0002**	0.788	High	0.586	Intermediate	0.280	Low	2/4
**02-0003**	0.725	High	0.400	Low	0.530	Intermediate	2/4
**02-0004**	0.406	Intermediate	0.415	Intermediate	0.681	High	1/4
**02-0005**	0.559	Intermediate	0.526	Intermediate	0.510	Intermediate	1/4
**02-0006**	0.341	Low	0.500	Intermediate	0.616	High	1/4
**02-0007**	0.564	Intermediate	0.479	Intermediate	0.600	Intermediate	1/4
**02-0008**	0.306	Low	0.502	Intermediate	0.639	High	1/4
**02-0009**	0.660	High	0.560	Intermediate	0.619	High	2/4
**02-0010**	0.491	Intermediate	0.558	Intermediate	0.548	Intermediate	1/4
**02-0011**	0.599	Intermediate	0.490	Intermediate	0.641	High	1/4
**02-0012**	0.749	High	0.615	High	0.373	Low	2/4
**02-0013**	0.787	High	0.745	High	0.311	Low	2/4

Supplementary Table S3. Number of patients classified as Low, Intermediate, and High for each immune target, based on tertile thresholds (33rd/66th percentile) applied to the 39-patient discovery cohort. The middle (Intermediate) tertile was excluded from classifier training (Section 2.8); Low and High cutoff values are shown for reference. Note the uneven split for CD8+ T cells (CIBERSORT), reflecting tied values near the tertile boundaries in that variable.

Target	N_total	Low(n)	Low(%)	Intermediate(n)	Intermediate(%)	High(n)	High(%)	Low_cutoff	High_cutoff
CD8+ T cells (CIBERSORT)	39	16	41	8	20.5	15	38.5	0.05	0.1
Total Immune Score (ESTIMATE)	39	13	33.3	13	33.3	13	33.3	603.4174	1303.092

Supplementary Figure 1.

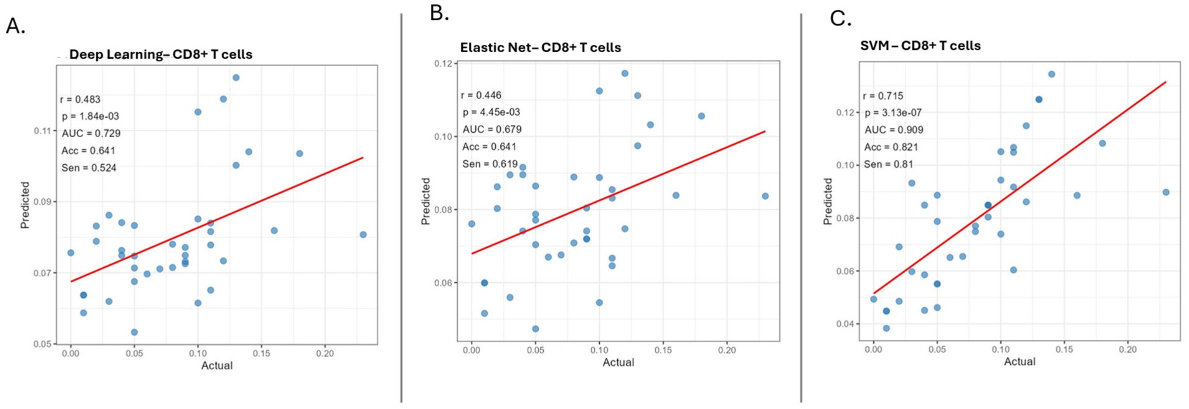

Figure S1. Correlation between actual and predicted immune scores—CD8+ T cell infiltration (CIBERSORT).
(A) Scatter plot of actual versus predicted CD8+ T cell infiltration scores across all 39 training patients, showing the linear fit (red line) with Pearson correlation coefficient and p-value. **(B)** Scatter plot restricted to the held-out test set (n = 7), showing predicted versus actual values for each model. **(C)** Per-model scatter plots comparing actual and predicted CD8+ T cell scores on the test partition, with individual regression lines and performance statistics annotated for Random Forest, SVM, Deep Learning, and Elastic Net.

Supplementary Figure 2.

Train (n = 32) (80%)
Test (n = 7) (20%)

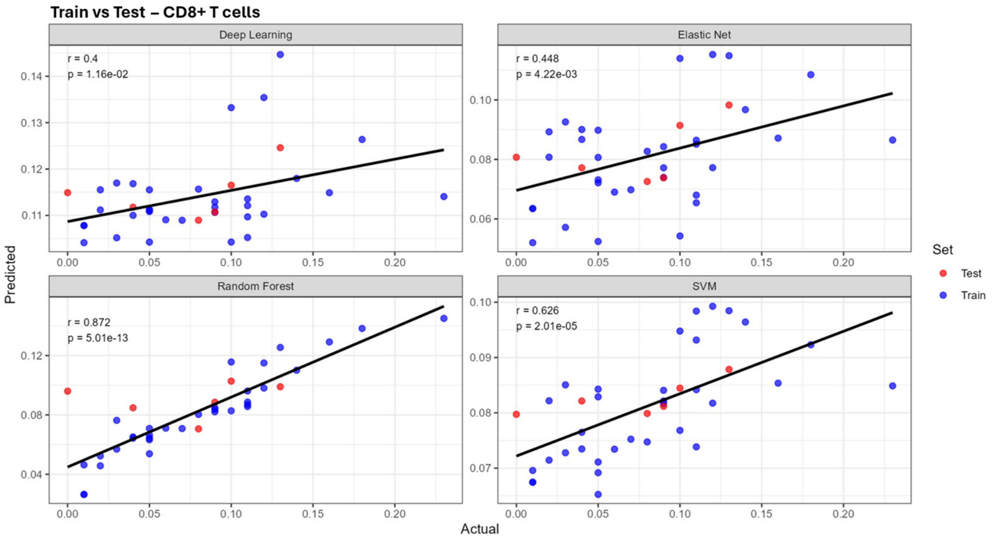

Figure S2. Train versus test set predicted probabilities—CD8+ T cell infiltration (CIBERSORT).
Scatter plot of actual versus predicted CD8+ T cell infiltration values for the full 39-patient cohort, stratified by training (n = 32, 80%) and test (n = 7, 20%) partitions. Training set predictions are shown in blue and test set predictions in red. The diagonal line represents perfect prediction. Model performance statistics are annotated in the upper left. Features used: 21 radiomic features.

Supplementary Figure 3.

Train (n = 25) (65%)
Test (n = 14) (35%)

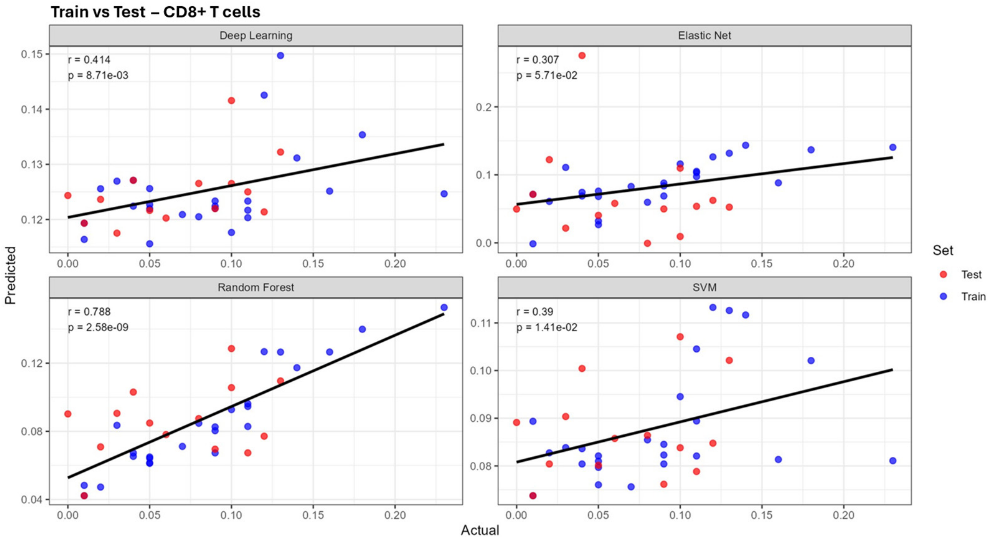

Figure S3. Train versus test set predicted values for CD8+ T cells across four machine learning models (Deep Learning, Elastic Net, Random Forest, SVM), secondary 65/35 split (n = 25 train, n = 14 test), assessing sensitivity-to-partition ratio.

Supplementary Figure 4.

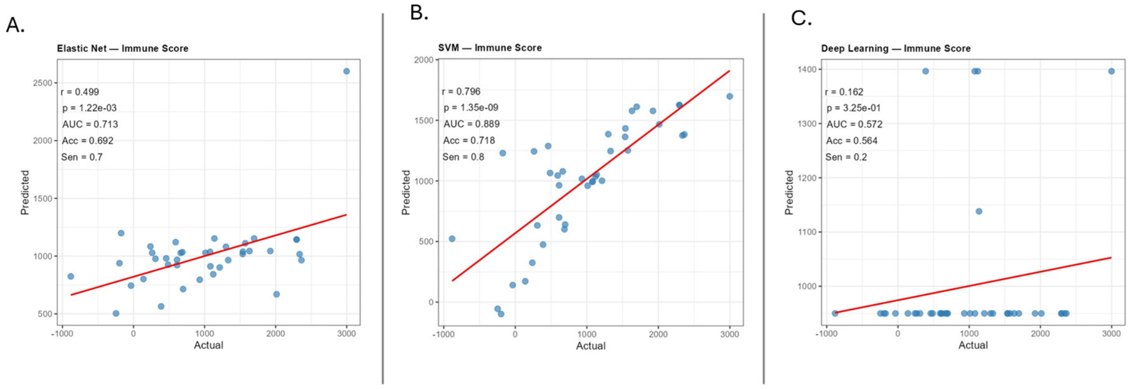

Figure S4. Correlation between actual and predicted immune scores—Total Immune Score (ESTIMATE).
(A) Scatter plot of actual versus predicted Total Immune Scores across all 39 training patients, with Pearson correlation and p-value annotated. **(B)** Scatter plot for the held-out test set (n = 7) showing per-model predicted versus actual Immune Scores. **(C)** Per-model scatter plots for the test partition with individual regression lines and performance statistics for Random Forest, SVM, Deep Learning, and Elastic Net.

Supplementary Figure 5.

Train (n = 32) (80%)
Test (n = 7) (20%)

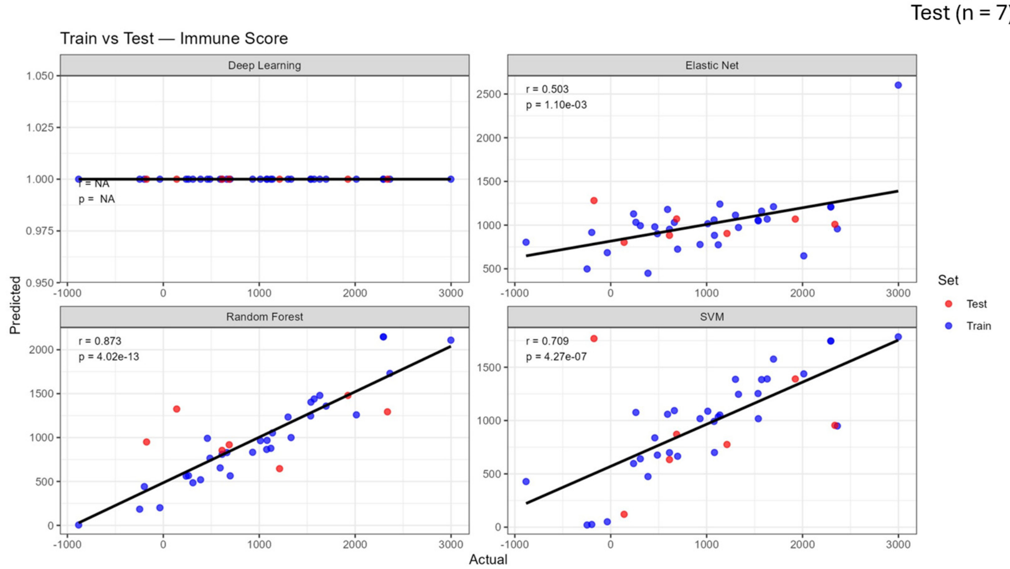

Figure S5. Train versus test set predicted probabilities—Total Immune Score (ESTIMATE).
Scatter plot of actual versus predicted Total Immune Score values for the full 39-patient cohort, stratified by training (n = 32, 80%) and test (n = 7, 20%) partitions. Training set predictions are shown in blue and test set predictions in red. The diagonal line represents perfect prediction. Model performance statistics are annotated in the upper left. Features used: 21 radiomic features.

Supplementary Figure 6.

Train (n = 25) (65%)
Test (n = 14) (35%)

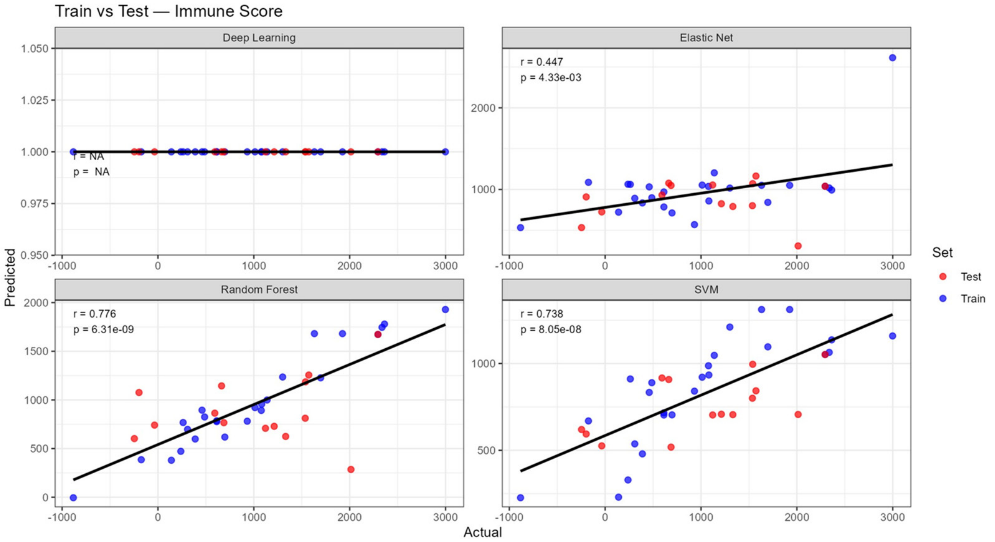

Figure S6. Train versus test set predicted values for Total Immune Score (ESTIMATE) across four machine learning models (Deep Learning, Elastic Net, Random Forest, SVM), secondary 65/35 split (n = 25 train, n = 14 test), assessing sensitivity-to-partition ratio.

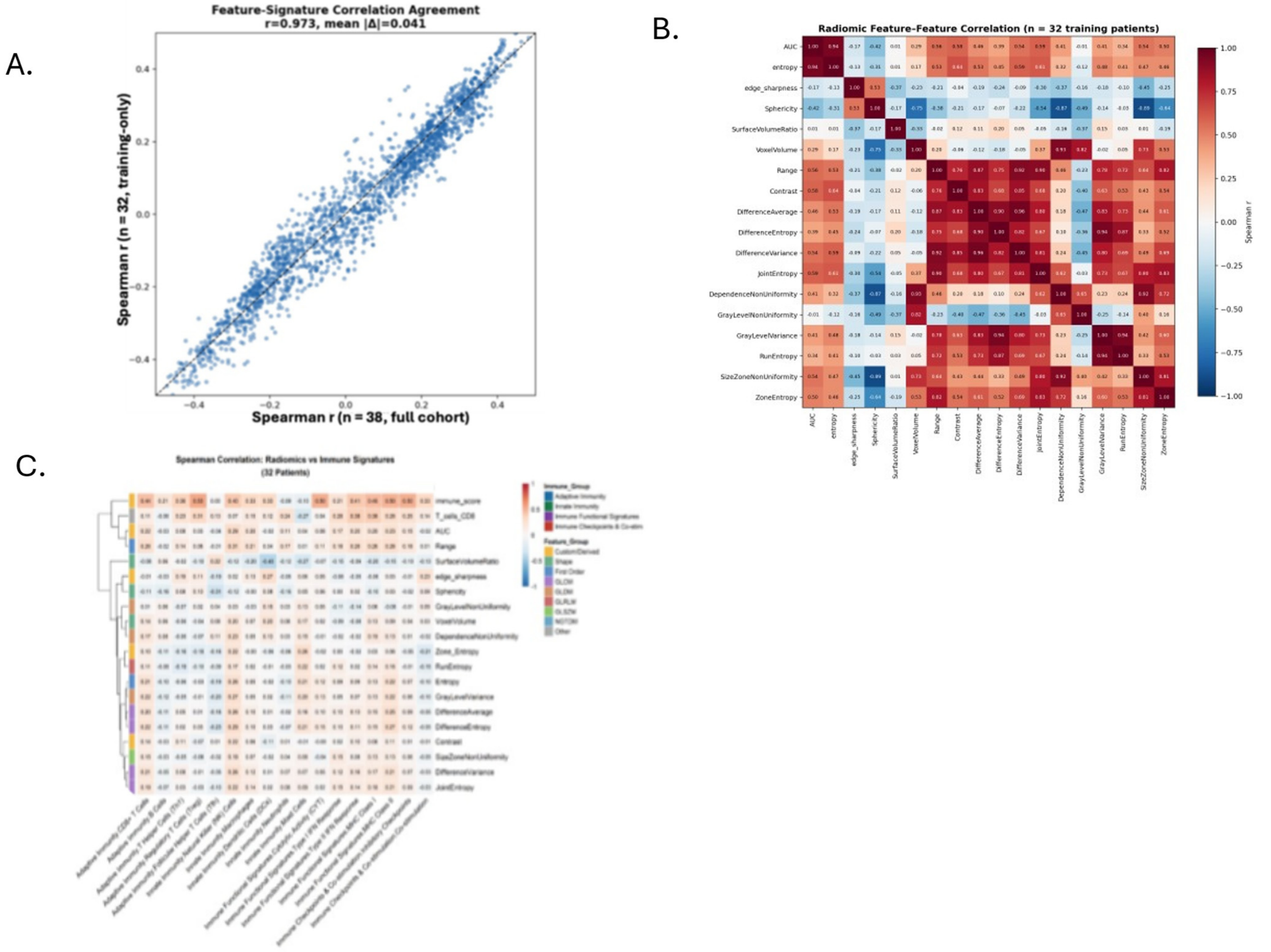

Figure S7. Feature-selection correlation stability at n = 32.

(A) Spearman correlation heatmap between radiomic features and the 17 immune gene signatures, computed using the 32-patient training partition only. **(B)** Agreement between the training-only (n = 32) and full-cohort (n = 38) correlation matrices, shown as a cell-by-cell scatter plot across all feature–signature pairs.
