# Supplementary material for: Evaluation of MRI Radiomic Features as Surrogates of Tumor Immune Status in Breast Cancer: A Pilot Study of MRI-Derived Radiomic Features for Non-Invasive Immune Profiling in Breast Cancer
Source: Cancers (Basel). 2026 Sep 23;18(19):3087. doi: 10.3390/cancers18193087 (PMC13664318; doi:10.3390/cancers18193087)
Supplement: Supplementary file 1 [file cancers-18-03087-s001.zip › File S1. RadioImmune tool.html]

RadioImmune — CD8+ T Cell Classifier


🧬

# RadioImmune

Radiomic-based CD8+ T cell infiltration classifier. Enter 18 radiomic features to predict CD8+ T cell immune status from imaging data.

Predict
How It Works

Quick Fill

Load a sample patient profile to see the classifier in action.

🔴 High — Patient A
🔴 High — Patient B
🔵 Low — Patient C
🔵 Low — Patient D
⚪ Cohort Mean
✕ Clear

Radiomic Feature Input

Enter all 18 radiomic feature values. Use the reference Excel sheet to look up values for each patient and confirm the prediction matches.

⚡ Classify Patient
Clear All

Running logistic approximation…

CD8+ T Cell Specific Score

CD8+ T Cell Infiltration

—

P(High CD8+ Infiltration)
—

Threshold
0.50

Low cutoff (≤33rd pct)
≤ 0.0500

High cutoff (≥66th pct)
≥ 0.1000

—

P(High)

Feature Importance — CD8+ T Cell Model

Random Forest mean decrease in impurity across 500 trees.

1

### 18 Radiomic Features Extracted

Imaging data is processed to extract 18 handcrafted radiomic descriptors spanning shape (Sphericity, VoxelVolume, SurfaceVolumeRatio), intensity (AUC, Range, Contrast), and texture features from GLCM, GLSZM, and GLRLM.

2

### Tertile-Based Binary Labelling

CD8+ T cell infiltration (CIBERSORT fraction) is binarised using tertile cutoffs. Patients at or below the 33rd percentile (≤ 0.05) are labelled **Low**; those at or above the 66th percentile (≥ 0.10) are labelled **High**. The middle tercile is excluded from training to maximise class separation.

3

### Random Forest Classification

A 500-tree Random Forest is trained on the 31 trainable patients (16 Low, 15 High) using all 18 standardised radiomic features. Feature importances reflect the mean decrease in impurity across all trees.

4

### Logistic Approximation for Browser Inference

Because Random Forests cannot run in the browser, a logistic regression is fitted to the same standardised training data. The logistic model captures the linear decision boundary and enables real-time inference. The probability threshold is fixed at 0.50. Feature values are standardised using the training set mean and standard deviation before prediction.

5

### Verification

Use the provided Excel reference sheet (*RadioImmune\_Patient\_Reference.xlsx*) to look up a patient by Sample ID, enter their radiomic feature values in this tool, and confirm the predicted class matches the reference classification.
